# An early prediction model for gestational diabetes mellitus based on genetic variants and clinical characteristics in China

**DOI:** 10.1186/s13098-022-00788-y

**Published:** 2022-01-24

**Authors:** Qi Wu, Yanmin Chen, Menglin Zhou, Mengting Liu, Lixia Zhang, Zhaoxia Liang, Danqing Chen

**Affiliations:** 1 Obstetrical Department, Women’s Hospital, School of Medicine, Zhejiang University, 1 Xueshi Road, Hangzhou, 310006 China; 2 Department of Epidemiology, School of Public Health and Tropical Medicine, Tulane University, New Orleans, Los Angeles United States of America

**Keywords:** Gestational diabetes mellitus, Single nucleotide polymorphism, Clinical characteristics, Prediction model, Early pregnancy

## Abstract

**Objectives:**

To evaluate the influence of genetic variants and clinical characteristics on the risk of gestational diabetes mellitus (GDM) and to construct and verify a prediction model of GDM in early pregnancy.

**Methods:**

Four hundred seventy five women with GDM and 487 women without, as a control, were included to construct the prediction model of GDM in early pregnancy. Both groups had complete genotyping results and clinical data. They were randomly divided into a trial cohort (70%) and a test cohort (30%). Then, the model validation cohort, including 985 pregnant women, was used for the external validation of the GDM early pregnancy prediction model.

**Results:**

We found maternal age, gravidity, parity, BMI and family history of diabetes were significantly associated with GDM (OR > 1; *P* < 0.001), and assisted reproduction was a critical risk factor for GDM (OR = 1.553, *P* = 0.055). *MTNR1B* rs10830963, *C2CD4A/B* rs1436953 and rs7172432, *CMIP* rs16955379 were significantly correlated with the incidence of GDM (AOR > 1, *P* < 0.05). Therefore, these four genetic susceptible single nucleotide polymorphisms (SNPs) and six clinical characteristics were included in the construction of the GDM early pregnancy prediction model. In the trial cohort, a predictive model of GDM in early pregnancy was constructed, in which genetic risk score was independently associated with GDM (AOR = 2.061, *P* < 0.001) and was the most effective predictor with the exception of family history of diabetes. The ROC-AUC of the prediction model was 0.727 (95% CI 0.690–0.765), and the sensitivity and specificity were 69.9% and 64.0%, respectively. The predictive power was also verified in the test cohort and the validation cohort.

**Conclusions:**

Based on the genetic variants and clinical characteristics, this study developed and verified the early pregnancy prediction model of GDM. This model can help screen out the population at high-risk for GDM in early pregnancy, and lifestyle interventions can be performed for them in a timely manner in early pregnancy.

**Supplementary Information:**

The online version contains supplementary material available at 10.1186/s13098-022-00788-y.

## Introduction

Gestational diabetes mellitus (GDM) is a common obstetric disease that affects nearly 7% of pregnant women and their offspring [1]. It is known to be associated with numerous adverse perinatal outcomes, such as gestational hypertension, eclampsia, abortion, preterm delivery, macrosomia, stillbirth, and others, which complicate 3% to 25% of pregnancies [2–4]. In addition, the incidence of type 2 diabetes (T2DM) for GDM women is as high as 50% to 70% during postpartum follow-up [5], making GDM a main source of T2DM in middle-aged women. More seriously, GDM can also have an impact on the long-term health of offspring, leading to a higher risk of obesity, hyperlipidemia, T2DM, hypertension, brain damage and neuropsychiatric disorders through genetic susceptibility, hyperglycemia, chronic inflammation and other comprehensive factors [6].

For these reasons, early prevention and treatment of GDM is critical for ameliorating both short-term and long-term consequences for mothers and offspring. However, the oral glucose tolerance test (OGTT) is currently used in the diagnosis of GDM during the second trimester (24–28 weeks) [7], when the adverse effects of hyperglycemia on mothers and fetuses has already occurred. Unfortunately, at present there is no authoritative high-risk screening for GDM in early pregnancy. The risk of GDM is determined by both genetic susceptibility and environmental factors. GDM and its secondary T2DM are complex polygenic diseases with the following characteristics: more than one gene is involved in pathogenesis, and each gene has a different degree of action [8]. The genome-wide association study (GWAS) identified various susceptibility loci for GDM and T2DM in different population [9–12]. Some single nucleotide polymorphisms (SNPs) have been found to be associated with susceptibility to GDM in Asian population [13], such as Korean [14] and Thai [15], but the results remained inconclusive in Chinese population. In addition, previous studies also indicated that women with advanced maternal age [16], obesity [17], sedentary lifestyle [18] were more likely to develop GDM. Therefore, we intended to find GDM-associated SNPs in Chinese population and hypothesized that an early pregnancy prediction model for GDM can be constructed using genetic risk variants and clinical factors.

To test this hypothesis, we performed a case–control study to assess the combined effects of genetic information and clinical factors for the early prediction of GDM and validated its predictive values in another prospective cohort.

## Methods

This study was conducted in the Women’s Hospital School of Medicine Zhejiang University, including pregnant women who underwent a 75-g OGTT at 24–28 gestational weeks and consented to DNA sampling at our center. According to the recommendations from the International Association of Diabetes and Pregnancy Study Groups (IADPSG) [7], women with any blood glucose value greater than the criteria (fasting blood glucose [FBG] 5.1 mmol/L, blood glucose after 1 h [1-h BG] 10.0 mmol/L, blood glucose after 2 h [2-h BG] 8.5 mmol/L) were diagnosed with GDM, while the rest formed a control group. Approval for this study was obtained from the hospital’s ethics committee (IRB-20200162-R).

We designed the early pregnancy prediction model for GDM using a two-phase approach, which included development and validation. During the development phase, we performed a case–control study with 500 GDM and 500 controls to initially establish the prediction model. Because clinical characteristics were indispensable in the model construction, we randomly selected pregnant women who came to the hospital at the same time period without matching. The development cohort was randomly divided into the trial cohort (70%) to construct the prediction model and the test cohort (30%) to internally validate the predictive effect of the model. Moreover, we established a validation cohort with another 1000 participants to externally verify the performance of the model.

We genotyped 16 SNPs previously reported to be significantly associated with GDM or T2DM, including variants in loci known to regulate insulin secretion and function in GDM (*MTNR1B*, *SLC30A8*, *CDKAL1*, etc.) and in loci associated with T2DM or GDM through other potential mechanisms (*C2CD4A/B*, *CMIP*, etc.) (See Additional file 1: Table S1 for details of the selection). Genotyping was performed by Mulitiplex Snapshot assays, and all genotype distributions did not deviate significantly from the Hardy–Weinberg equilibrium (*P* ≥ 0.05) (Additional file 1: Table S2). In our study, of the 16 SNPs evaluated, 4 SNPs (rs10830963 in *MTNR1B*, rs1436953 and rs7172432 in *C2CD4A/B*, rs16955379 in *CMIP*) were found to be associated with the risk of GDM in at least one genetic model (Additional file 1: Tables S3 and 4), and so they were included in subsequent analyses. Pregnant women missing information for those four SNPs were excluded from the prediction model.

The clinical characteristics of early pregnancy were gathered from medical records, such as maternal age, gravidity, parity, height (self-reported at first prenatal care), pre-pregnancy weight (self-reported at first prenatal care), body mass index (BMI was calculated by weight and height), the way of conception, family history, previous medical history, and others. Pregnant women with missing or abnormal clinical information were excluded, as were those with pre-pregnancy conditions such as diabetes, hypertension, or other vital organ diseases.

Blood glucose, insulin levels and glycosylated hemoglobin (HbA1c) were tested in the biochemical laboratory of the Women’s Hospital School of Medicine Zhejiang University at 24–28 gestational weeks. Homeostatic model assessments of islet β-cell function (HOMA-β) and insulin resistance (HOMA-IR) were calculated by FBG and fasting insulin levels.

Assuming an additive genetic model, the genotypes were scored as 0, 1, and 2 for each risk allele, and the individual effect of each SNP on the risk of GDM were evaluated by logistic regression analysis with or without adjustment of clinical characteristics. Multiple linear regression analysis with adjustment for maternal age, gravidity, parity, BMI, family history of diabetes and the way of conception was performed to explore the relationship between each SNP and continuous variables (e.g., blood glucose, insulin levels, HbA1c, HOMA). Bonferroni correction was used to counteract the problem of multiple comparisons. The genetic risk score (GRS) was counted to clarify the combined effect of four SNPs on the risk of GDM, which was calculated by single-SNP logistic regression analysis to better assess the genetic effect of each SNPs. The clinical characteristics between the GDM and control groups were compared by t test or χ^2^ test. The prediction model was constructed by logistic regression analysis. Receiver operating characteristic (ROC) -area under the curve (AUC) was calculated to evaluate the predictive powers. Statistical analyses were performed using SPSS version 20.0. *P* < 0.05 was considered statistically significant.

## Results

### Inclusion of model factors in the development phase

**Fig. 1:**
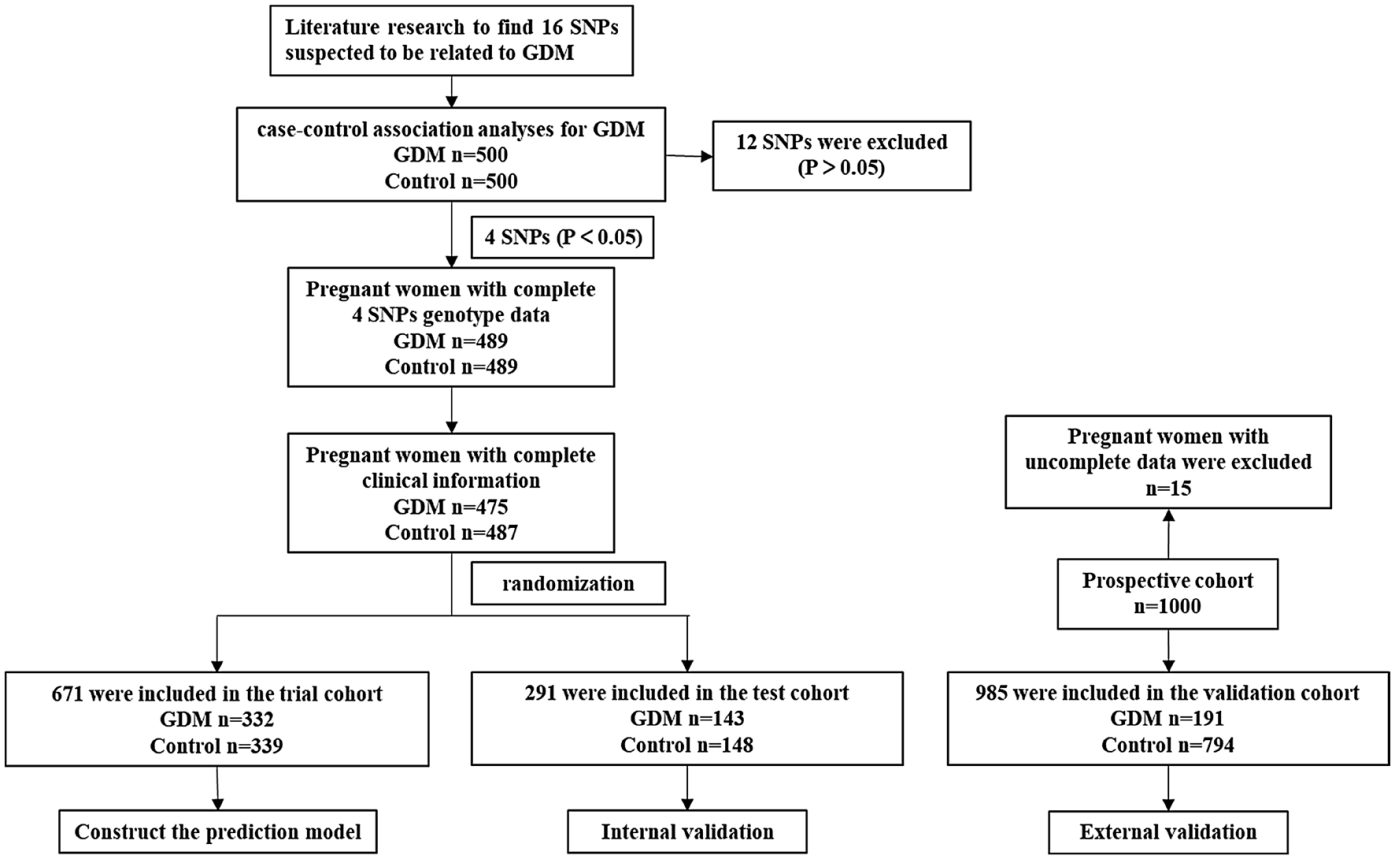
Flow diagram of the selection of cases of GDM and controls in the development and validation phase

Among Chinese pregnant women, 475 GDM and 487 controls with complete four SNPs genotype data and clinical information were included in the analysis of the development cohort (Fig. 1). In general, pregnant women with GDM had higher maternal age, gravidity, parity, BMI than the controls, so did the rate of family history of diabetes (all *P* < 0.001). We also noticed assisted reproduction was more likely in GDM, although it did not achieve statistical significance (7.2% vs 10.7%, *P* = 0.054). As expected, the blood glucose, insulin and HbA1c levels were significantly higher in GDM compared to the controls (all *P* < 0.001). Detailed information was supplied in Additional file 1: Table S5.

We performed a univariate statistical analysis of the correlation between the clinical characteristics of early pregnancy and GDM. The results showed that maternal age, gravidity, parity, BMI, family history of diabetes were significantly associated with the increased risk of GDM (OR > 1; *P* < 0.001). Stratified analysis revealed that the risk of GDM increased with maternal age, especially in pregnant women over 40 years old whose risk of GDM was 18.79 times higher than women under 25. Women with more than two previous pregnancies were more likely to develop GDM than women pregnant for the first time (OR 2.509, 95% CI 1.837–3.428). In addition, overweight was a risk factor for GDM (OR 3.745, 95% CI 2.377–5.901), while underweight was a protective factor (OR 0.684, 95% CI 0.472–0.993). As for assisted reproduction, it also tended to increase the risk of GDM, which was borderline-significant. Thus, these six clinical characteristics were incorporated into the prediction model of GDM (Table 1).

**Table 1:** Association of clinical characteristics with GDM in the Chinese population

	The proportion of GDM^a^	OR^b^ (95% CI)	*P*
Maternal age (years)		1.174 (1.136–1.213)	< 0.001
< 25	4 (15.4)	–	–
25–29	91 (32.7)	2.676 (0.896–7.996)	0.078
30–34	190 (49.9)	5.471 (1.850–16.177)	0.002
35–39	149 (66.5)	10.927 (3.634–32.855)	< 0.001
≥ 40	41 (77.4)	18.792 (5.414–65.230)	< 0.001
Gravidity		1.397 (1.248–1.565)	< 0.001
1	152 (41.3)	–	–
2	127 (44.3)	1.128 (0.826–1.541)	0.449
≥ 3	196 (63.8)	2.509 (1.837–3.428)	< 0.001
Parity		1.686 (1.334–2.133)	< 0.001
Nulliparous	218 (42.1)	–	–
Multiparous	257 (57.9)	1.891 (1.463–2.445)	< 0.001
Pre-pregnancy BMI (kg/m^2^)		1.205 (1.148–1.265)	< 0.001
Normal (18.5–24.9 kg/m^2^)	53 (37.9)	–	–
Underweight (< 18.5 kg/m^2^)	332 (47.1)	0.684 (0.472–0.993)	< 0.001
Overweight (≥ 25 kg/m^2^)	90 (76.9)	3.745 (2.377–5.901)	< 0.001
Family history of diabetes			
No	439 (47.8)	-	–
Yes	36 (81.8)	4.910 (2.258–10.679)	< 0.001
Way of conception			
Natural reproduction	424 (48.4)	–	–
Assisted reproduction	51 (59.3)	1.553 (0.990–2.437)	0.055

^a^ The proportion of GDM was expressed as number (percentage)

^b^ Univariate logistic regression in the Chinese population

The four selected SNPs, rs10830963 in *MTNR1B*, rs1436953 and rs7172432 in *C2CD4A/B*, rs16955379 in *CMIP* were significantly related to GDM with or without adjustment for clinical information. Among them, rs10830963 had the strongest relationship with GDM (adjusted OR 1.387, 95% CI 1.136–1.694), while rs1436953, rs7172432, rs16955379 were found to have a 1.257-fold, 1.308-fold and 1.291-fold increased risk of GDM, respectively (Table 2). Collinearity diagnosis suggested that there was no collinearity among the four SNPs (Additional file 1: Table S6). Moreover, the *G* allele of rs10830963 was correlated with elevated 1-h OGTT BG (β = 0.286, *P* = 0.002) and HOMA-IR (β = 0.162, *P* = 0.017), while the *T* allele of rs1436953 was associated with increased 2-h OGTT BG (β = 0.222, *P* = 0.010) and HOMA-IR (β = 0.175, *P* = 0.011). We also observed the association of rs7172432 with higher 1-h OGTT BG (β = 0.214, *P* = 0.025), 2-h OGTT BG (β = 0.242, *P* = 0.005), HbA1c (β = 0.036, *P* = 0.038), HOMA-IR (β = 0.157, *P* = 0.020). The *C* allele of rs16955379 also had the similar effect on FBG (β = 0.055, *P* = 0.035), 1-h OGTT BG (β = 0.236, *P* = 0.018) and 2-h OGTT BG (β = 0.201, *P* = 0.025). Nevertheless, the difference was no longer significant after Bonferroni correction, except for the *G* allele of rs10830963 and 1-h OGTT BG, and the *G* allele of rs7172732 and 2-h OGTT BG (Table 3).

**Table 2:** Single-SNP association analysis of GDM

Closest gene	SNP	A/a	Control	GDM	OR (95% CI)	*P*	aOR^a^ (95% CI)	β^b^	*P*
			AA/Aa/aa	AA/Aa/aa					
*MTNR1B*	rs10830963	C/**G**	161/239/87	118/246/111	1.327 (1.105–1.592)	0.002	1.387 (1.136–1.694)	0.327	0.001
*C2CD4A/B*	rs1436953	C/**T**	236/205/46	194/218/63	1.292 (1.068–1.562)	0.008	1.257 (1.021–1.548)	0.229	0.031
*C2CD4A/B*	rs7172432	A/**G**	203/227/57	161/242/72	1.283 (1.062–1.551)	0.010	1.308 (1.061–1.611)	0.268	0.012
*CMIP*	rs16955379	**C**/T	250/189/48	268/175/32	1.220 (1.001–1.486)	0.048	1.291 (1.039–1.605)	0.256	0.021

A/a, major allele/minor allele; Risk allele was underlined and in bold

^a^The analysis was adjusted for maternal age, gravidity, parity, BMI, family history of diabetes and way of conception

^b^Regression coefficients in the multivariable logistic regression analysis

**Table 3:** Association of 4 SNPs with glucose metabolism-related indicators

SNP	A/a	FBG		1-h OGTT BG		2-h OGTT BG		HbA1c		HOMA-β		HOMA-IR	
		β (95% CI)	*P*	β (95% CI)	*P*	β (95% CI)	*P*	β (95% CI)	*P*	β (95% CI)	*P*	β (95% CI)	*P*
rs10830963	C/G	0.04 (− 0.01–0.09)	0.077	0.29 (0.11–0.47)	0.002	0.13 (− 0.04–0.29)	0.126	0.01 (− 0.03–0.04)	0.717	3.20 (− 40.56–46.96)	0.886	0.16 (0.03–0.29)	0.017
rs1436953	C/T	0.03 (− 0.02–0.08)	0.257	0.19 (0.00–0.38)	0.051	0.22 (0.05–0.39)	0.010	0.03 (0.00–0.07)	0.051	− 3.06 (− 47.48–41.36)	0.892	0.18 (0.04–0.31)	0.011
rs7172432	A/G	0.01 (− 0.04–0.06)	0.720	0.21 (0.03–0.40)	0.025	0.24 (0.07–0.41)	0.005	0.04 (0.00–0.07)	0.038	− 12.69 (− 56.45–31.08)	0.569	0.16 (0.02–0.29)	0.020
bs16955379	C/T	0.06 (0.00–0.11)	0.035	0.24 (0.04–0.43)	0.018	0.20 (0.03–0.38)	0.025	0.03 (− 0.01–0.06)	0.147	− 5.180 (− 50.25–39.90)	0.821	0.06 (− 0.08–0.19)	0.432

A/a: major allele/minor allele; Risk allele was underlined and in bold. FBG: fasting blood glucose; 1-h OGTT BG: OGTT blood glucose after 1 h; 2-h OGTT BG: OGTT blood glucose after 2 h; HbA1c: glycosylated hemoglobin; HOMA-β: homeostatic model assessments of islet β-cell function; HOMA-IR: homeostatic model assessments of insulin resistance

All analyses were adjusted for maternal age, gravidity, parity, BMI, family history of diabetes and way of conception in the linear regression model

### Model construction and internal validation in the development phase

**Fig. 2:**
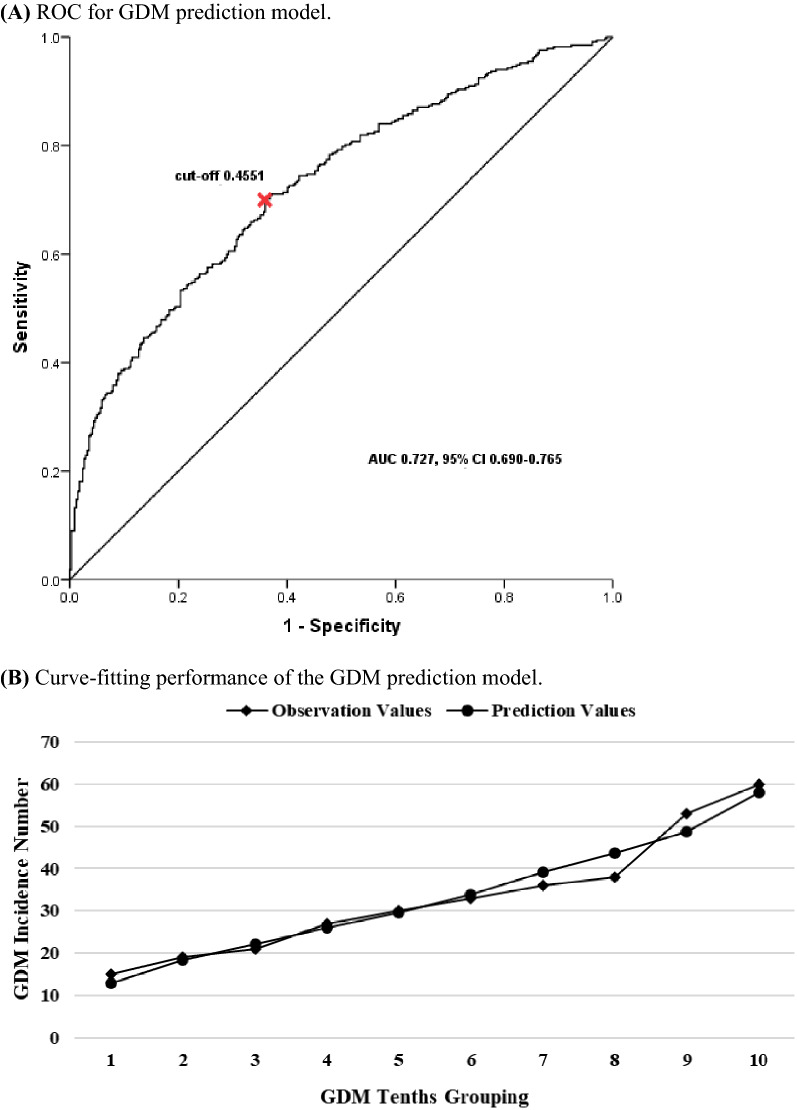
The performance of the GDM prediction model with GRS and clinical characteristics

According to the above results, we planned to establish the GDM prediction model in the trial cohort, including maternal age, gravidity, parity, pre-pregnancy BMI, family history of diabetes, the way of conception and GRS that was calculated to clarify the combined effect of the four SNPs on the risk of GDM. In the model, we found that GRS was independently correlated with GDM (aOR 2.061, 95% CI 1.382–3.073), which was the most effective predictor, with the exception of family history of diabetes (aOR 4.133, 95% CI 1.613–10.585) (Table 4). The ROC-AUC of the prediction model with GRS and clinical characteristics was 0.727 (95% CI 0.690–0.765), and the sensitivity and specificity were 69.9% and 64.0%, respectively. In addition, the Hosmer–Lemeshow test showed that the model had a good calibration ability (χ^2^ = 5.141, *P* = 0.742) (Fig. 2).

**Table 4:** Prediction model for GDM constructed in the trial cohort

Model	OR^a^	*P*	aOR^b^	β	Wals	*P*
GRS	1.855 (1.287–2.674)	0.001	2.061 (1.382–3.073)	0.723	12.598	< 0.001
Maternal age	1.150 (1.107–1.194)	< 0.001	1.133 (1.082–1.187)	0.125	27.879	< 0.001
Gravidity	1.364 (1.194–1.559)	< 0.001	1.089 (0.896–1.323)	0.085	0.734	0.392
Parity	1.712 (1.291–2.270)	< 0.001	0.957 (0.629–1.457)	− 0.044	0.042	0.838
Pre-pregnancy BMI	1.198 (1.131–1.269)	< 0.001	1.167 (1.100–1.239)	0.155	25.936	< 0.001
Family history of diabetes	4.520 (1.829–11.165)	0.001	4.133 (1.613–10.585)	1.419	8.743	0.003
Way of conception	1.596 (0.930–2.740)	0.090	1.140 (0.619–2.098)	0.131	0.176	0.675

^a^Univariate logistic regression in the trial cohort

^b^The prediction model was constructed by multivariate logistic regression

We then evaluated the predictive power of the constructed model in the test cohort. The ROC-AUC in the test cohort was 0.776 (95% CI 0.722–0.830), and the sensitivity and specificity were respectively 71.3% and 75.0%. The Hosmer–Lemeshow test showed that there was no significant difference between the fitted values and the observed values among GDM groups (χ^2^ = 6.637, *P* = 0.576) (Additional file 1: Fig. S1A).

### External validation in the validation phase

In the analysis of the validation cohort, there were 985 participants with complete four SNP-genotype data and clinical information, including 191 GDM and 794 controls, with a 19.4% incidence of GDM (Fig. 1). The clinical characteristics were similar to those found in the development cohort (Additional file 1: Table S5). The prediction model was externally verified in the validation cohort, whose ROC-AUC was 0.620 (95% CI 0.573–0.667) with a sensitivity of 52.4% and a specificity of 68.8%. The Hosmer–Lemeshow test also showed the good calibration ability of the GDM prediction model (χ^2^ = 10.741, *P* = 0.217) (Additional file 1: Fig. S1B).

## Discussion

Based on genetic variants and clinical characteristics in early pregnancy, this study constructed a prediction model of GDM in a two-phase approach. In this model, a total of 4 SNPs and 6 basic clinical features were included, which were closely related to the risk of GDM in Chinese pregnant women. The SNPs can be determined by genotyping from peripheral blood of pregnant women in early pregnancy. Clinical features can be simply obtained from the medical records in the first prenatal examination. By inputting these data, the model can screen out women at high risk of GDM early in pregnancy in order to facilitate a timely intervention.

In this study, we observed that the *G* allele of rs10830963 in *MTNR1B* was significantly associated with the risk of GDM, which increased 1.387 times for each additional risk allele. Previous studies found similar results: polymorphism at rs10830963 was a specific genetic factor for GDM [19, 20]. In fact, *MTNR1B* was strongly expressed in islet β cells and maintained glucose homeostasis by regulating insulin release [21]. Our results suggested that the *G* allele of rs10830963 was independently associated with increased 1-h OGTT BG and insulin resistance, which also supported the possibility of this mechanism from an epidemiological perspective. Although the molecular mechanism of *C2CD4A/B* in regulating glucose homeostasis has not been well-characterized, the association of risk alleles in *C2CD4A/B* with T2DM has been repeatedly demonstrated in several studies [22–24]. In our study, two loci of *C2CD4A/B* were first identified to be associated with GDM in Chinese pregnant women. Women with *T* alleles in rs1436953 and *G* alleles in rs7172432 were more susceptible to GDM. Furthermore, our study on relationship between *C2CD4A/B* variants and glucose metabolism-related indicators suggested that *C2CD4A/B* might be related to islet dysfunction in GDM, which was not surprising given the similarities in the pathogenesis and epidemiology of T2DM and GDM. Interestingly, this study also found for the first time a significant association between *CMIP* rs16955379 and GDM. Previous studies suggested that *CMIP* was associated with lipid metabolism, and its rs16955379 variant was linked to lipid metabolism disorders [25], which might thus increase the risk of T2DM [26]. Cho et al. [11] found *C* alleles in rs16955379 was a risk factor for T2DM in East Asian populations. More importantly, our study also showed that the *C* allele of rs16955379 could increase the levels of FBG, 1-h OGTT BG and 2-h OGTT BG in pregnant women, which concurred with previous studies [27].

In addition to genetic variants, clinical characteristics also played a significant role in the incidence of GDM. Advanced maternal age has always been a high risk factor for GDM. Khali et al. [28] found that the incidence of GDM was positively correlated with maternal age and reached a peak at the age of 40. We also found pregnant women with multiple gravidities or parities were more likely to suffer from GDM, which has been confirmed by previous studies [29, 30]. Further, higher pre-pregnancy BMI was also a common risk factor for GDM. Obesity not only greatly increased the risk of GDM, but also made fetal congenital abnormalities, preterm delivery and even death more likely to occur [31]. Family history of diabetes was a clinical characteristic most strongly associated with GDM, and it increased the risk of GDM by more than 4 times in our study. This result was also reported by Harder et al. [32], and they found the influence of maternal diabetes on pregnant women was stronger than that of paternal diabetes. Besides, although no significant effect of assisted reproduction on the incidence of GDM was found in our study, it was still included in the construction of the prediction model. The reason for this was that current evidence indicated assisted reproduction could increase the risk of GDM [33, 34], and the experience of clinicians has also suggested pregnant women with assisted reproduction need more strict management to prevent GDM than those who conceive naturally.

In summary, we used the trial cohort to construct a prediction model of GDM in early pregnancy based on genetic variants and clinical characteristics. The prediction efficiency reached 0.727 with sensitivity and specificity of 69.9% and 64.0%, respectively. We then used the test cohort to primarily verify the model and found that the prediction model still had good model-discrimination ability. However, considering that internal validation often overestimates prediction accuracy, and we needed to determine whether the prediction model is valid for clinical applications, we prospectively collected validation cohort to conduct an external validation of the prediction model. The results of external validation showed that the model prediction efficiency did not become more ideal, but it still had a certain capability for model discrimination. Although model sensitivity was reduced, the model specificity remained excellent, reaching 68.8%. This means that the model can effectively exclude most non-GDM patients, and the others who may at high risk of GDM can be reduced by timely and effective lifestyle intervention and management.

As a pregnancy complication that seriously threatens the health of both mother and child, screening and diagnosis methods for GDM have long been a focus of research. All involved professional organizations recommend that pregnant women should take the OGTT at 24–28 gestational weeks as the gold standard for diagnosing GDM [35], but undertaking it is not advised for pregnant women in the early stage of pregnancy. This may be due to the fact that abnormal blood glucose detected thus may have existed before pregnancy and therefore cannot be clearly diagnosed as typical GDM that develops during pregnancy [36]. Initially, the IADPSG also proposed to use the FBG threshold of > 92 mg% as the diagnostic method for GDM before 24 gestational weeks. However, this was ultimately removed from the recommendation due to the lack of evidence for a convincing threshold in early pregnancy [37]. Instead of focusing on OGTT and blood glucose screening, we innovatively established an early pregnancy prediction model using genetic variants and clinical characteristics, which could be used to screen pregnant women at high risk of GDM in early pregnancy. It was the first advantage of our study. Many researchers have explored GDM prediction models in the past, but most of them were based on clinical features. Pan et al. [38] explored the combined predictive effect of pre-pregnancy BMI and first-trimester FBG on GDM. Sweetin et al. [39] developed a prediction model for early pregnancy on the basis of maternal lipid metabolites and clinical data. These studies ignored the important role of genetic factors in the incidence of GDM. Kawai et al. [40] explored the relationship between common T2DM risk SNPs and GDM, but their results were not prospectively verified in a new pregnant-women population, as it was in our study. The rigorous process of model predictor inclusion, construction and validation of the model was the second advantage of this study. Because GDM was a complex polygenic disease, we used GRS to comprehensively evaluate the combined effect of four SNPs on the risk of GDM. The results showed that GRS was strongly associated with an increased risk of GDM and was one of the most effective predictors in the prediction model. It was worth noting that we did not simply calculate the GRS by adding up each risk allele, but weighted each SNP according to its own correlation with GDM, so as to combine the genetic susceptibility of each SNPs more effectively. While focusing on the combined genetic effect of GDM, we did not ignore the specificity of each SNPs, which was also one of the advantages.

There were still some limitations. First, this was a regional single-center study from a large specialized hospital, whose population had a higher overall risk and could only represent pregnant women in the surrounding area of the hospital. Thus, it might not be fully applicable to all the population. However, all participants in our study were strictly grouped and analyzed to ensure the reliability of the research results. Second, this kind of study also leaded to a small sample size, which might affect its accuracy and reliability to some extent. Large-scale multicenter studies need to be performed to further verify the prediction model for GDM. Third, although we adjusted gravidity and parity as confounding factors in the analysis of GDM genetic susceptibility, we could not ignore the possibility that normal control pregnant women would develop GDM in future pregnancies, which might lead to grouping error and an underestimation for the effect of genetic factors on the risk of GDM.

In conclusion, an early pregnancy prediction model of GDM based on genetic variants and clinical characteristics was developed and verified in our study. This prediction model can screen in the first trimester for pregnant women at high risk for GDM, allowing physicians to start lifestyle and dietary interventions early so that women can maintain normal blood glucose levels during pregnancy. It is expected to fundamentally reduce the incidence of GDM and improve the quality of the newborn population.

## Supplementary Information

**Additional file 1**. Additional tables and figures.

## Data Availability

Some or all datasets generated during and/or analyzed during the current study are not publicly available but are available from the corresponding author on reasonable request.
