# Supplementary material for: An early prediction model for gestational diabetes mellitus based on genetic variants and clinical characteristics in China
Source: Diabetol Metab Syndr. 2022 Jan 24;14:15. doi: 10.1186/s13098-022-00788-y (PMC8785509; doi:10.1186/s13098-022-00788-y)
Supplement: Supplementary file 1 — Additional file 1. Additional tables and figures. [file 13098_2022_788_MOESM1_ESM.docx]

We reviewed previous literatures and selected candidate SNPs according to the pathogenesis of GDM, and the specific inclusion criteria were as follows:

(1) SNPs were involved in the main pathogenic mechanism, such as insulin secretion regulation and islet β-cell function. And SNPs related to lipid metabolism were also included because obesity also plays an important role in the complex pathogenesis of GDM.

(2) SNPs proved to be significantly correlated with GDM by previous studies were preferred to be included.

(3) Due to the similarities between the pathogenesis and genetic tendency of GDM and type 2 diabetes, SNPs found in previous studies that were significantly associated with type 2 diabetes were also included. Among them, SNPs with extensive studies, high effect values or Asian population as the research object were given priority.

Finally, 16 candidate SNPs of GDM susceptibility gene were selected in this study, as detailed in Supplemental Table 1.

**Table S1** Candidate SNPs of GDM susceptibility genes.

| Closest gene | Gene function | SNP | OR (95% CI) | Reference |
| --- | --- | --- | --- | --- |
| *MTNR1B* | Circadian regulation of insulin secretion | rs10830963 | 1.28 (1.05-1.55) | [1],[2],[3],[4] |
| *C2CD4A/B* | Regulate β cells proliferation | rs1436953 | 1.11 (1.07-1.16) | [5],[6] |
|  |  | rs7172432 | 1.12 (1.08-1.16) | [5],[7] |
| *CMIP* | Related to lipid metabolism | rs16955379 | 1.08 (1.05-1.12) | [6],[8] |
| *FADS1* | Polyunsaturated fatty acid metabolism | rs174550 | 1.66 (1.23-2.25) | [9] |
| *GLIS3* | Involved in β cell development | rs7041847 | 1.13 (1.05-1.20) | [7],[8],[10] |
|  |  | rs7875253 | 1.23 (1.10‐1.38) | [11] |
| *CDKAL1* | Degeneration of β cells and Regulation of insulin secretion | rs7756992 | 1.39 (1.20-1.61) | [12],[13],[14] |
|  |  | rs9465871 | 1.41 (1.21-1.66) | [15] |
|  |  | rs7754840 | 1.40 (1.13-1.72) | [1],[15],[16],[17] |
| *PEPD* | Related to lipid metabolism | rs3786897 | 1.11 (1.01-1.22) | [6],[7],[8] |
| *IGF2BP2* | Regulation of insulin-like growth factor translation | rs4376068 | 1.23 (1.15-1.32) | [18] |
| *SLC30A8* | Maturation and secretion of insulin | rs3802177 | 1.25 (1.07-1.46) | [12] |
|  |  | rs13266634 | 1.24 (1.07-1.43) | [19],[20],[21],[22] |
| *ADIPOQ* | Code for adiponectin | rs266729 | 1.56 (1.10-2.23) | [23],[24] |
| *TCF7L2* | Regulation of glucagon-like peptides | rs290487 | 1.25 (1.08-1.46) | [25] |

**Table S2** Hardy-Weinberg equilibrium for candidate SNPs in the Chinese population.

| Closest gene | SNP | A/a | χ^2^ | *P* |
| --- | --- | --- | --- | --- |
| *MTNR1B* | rs10830963 | C/**G** | 0.271 | 0.873 |
| *C2CD4A/B* | rs1436953 | C/**T** | 0.015 | 0.992 |
|  | rs7172432 | A/**G** | 0.787 | 0.675 |
| *CMIP* | rs16955379 | **C**/T | 0.815 | 0.665 |
| *FADS1* | rs174550 | T/**C** | 0.567 | 0.753 |
| *GLIS3* | rs7041847 | G/**A** | 0.129 | 0.938 |
|  | rs7875253 | **A**/C | 0.322 | 0.851 |
| *CDKAL1* | rs7756992 | **G**/A | 1.365 | 0.505 |
|  | rs9465871 | **C**/T | 1.387 | 0.500 |
|  | rs7754840 | G/**C** | 0.233 | 0.890 |
| *PEPD* | rs3786897 | **A**/G | 1.182 | 0.554 |
| *IGF2BP2* | rs4376068 | A/**C** | 0.253 | 0.881 |
| *SLC30A8* | rs3802177 | **G**/A | 0.088 | 0.957 |
|  | rs13266634 | **C**/T | 0.005 | 0.998 |
| *ADIPOQ* | rs266729 | **C**/G | 0.235 | 0.889 |
| *TCF7L2* | rs290487 | T/**C** | 0.005 | 0.997 |

A/a, major allele/minor allele; Risk allele was underlined and in bold.

**Table S3** Association analysis of candidate SNPs for GDM in the Chinese population.

| Closest gene | SNP | A/a | Control | GDM |  | Genotype-specific^a^ |  | Allele-specific | |
| --- | --- | --- | --- | --- | --- | --- | --- | --- | --- |
|  |  |  | AA/Aa/aa | AA/Aa/aa |  | *P* |  | OR (95%CI) | *P* |
| *MTNR1B* | rs10830963 | C/**G** | 164/245/87 | 122/258/115 |  | **0.006** |  | **1.329 (1.114-1.587)** | **0.002** |
| *C2CD4A/B* | rs1436953 | C/**T** | 238/211/46 | 201/228/67 |  | **0.022** |  | **1.303 (1.081-1.571)** | **0.006** |
|  | rs7172432 | A/**G** | 207/232/57 | 167/251/74 |  | **0.027** |  | **1.273 (1.061-1.528)** | **0.009** |
| *CMIP* | rs16955379 | **C**/T | 257/190/48 | 279/185/32 |  | 0.124 |  | 1.212 (0.994-1.479) | 0.058 |
| *FADS1* | rs174550 | T/**C** | 151/237/108 | 166/233/92 |  | 0.368 |  | 0.878 (0.735-1.049) | 0.152 |
| *GLIS3* | rs7041847 | G/**A** | 131/254/114 | 126/251/118 |  | 0.919 |  | 1.036 (0.869-1.236) | 0.690 |
|  | rs7875253 | **A**/C | 234/219/44 | 256/203/36 |  | 0.303 |  | 1.162 (0.957-1.410) | 0.128 |
| *CDKAL1* | rs7756992 | **G**/A | 123/275/100 | 138/248/112 |  | 0.230 |  | 1.012 (0.849-1.207) | 0.893 |
|  | rs9465871 | **C**/T | 126/274/94 | 132/241/114 |  | 0.127 |  | 0.946 (0.792-1.129) | 0.537 |
|  | rs7754840 | G/**C** | 158/252/88 | 171/243/82 |  | 0.642 |  | 0.923 (0.773-1.103) | 0.380 |
| *PEPD* | rs3786897 | **A**/G | 143/267/87 | 144/250/104 |  | 0.354 |  | 0.937 (0.785-1.118) | 0.468 |
| *IGF2BP2* | rs4376068 | A/**C** | 283/183/30 | 276/175/35 |  | 0.760 |  | 1.039 (0.846-1.275) | 0.716 |
| *SLC30A8* | rs3802177 | **G**/A | 148/255/92 | 171/238/88 |  | 0.312 |  | 1.116 (0.934-1.333) | 0.226 |
|  | rs13266634 | **C**/T | 151/254/93 | 171/234/90 |  | 0.350 |  | 1.101 (0.922-1.315) | 0.288 |
| *ADIPOQ* | rs266729 | **C**/G | 273/190/34 | 258/207/32 |  | 0.545 |  | 0.935 (0.766-1.141) | 0.509 |
| *TCF7L2* | rs290487 | T/**C** | 202/220/73 | 179/248/69 |  | 0.204 |  | 1.086 (0.906-1.302) | 0.373 |

A/a, major allele/minor allele; Risk allele was underlined and in bold.

^a^ *P* values were calculated by comparing three genotype groups using χ^2^ test.

**Table S4** Effects of susceptible genotypes on the risk for GDM under different genetic models.

| SNP | Genotypes |  | Additive genetic model  (**aa** vs AA) | |  | Dominant genetic model  (aa+Aa vs AA) | |  | Recessive genetic model  (aa vs Aa+AA) | |
| --- | --- | --- | --- | --- | --- | --- | --- | --- | --- | --- |
|  | AA/Aa/aa |  | OR (95%CI) | *P* |  | OR (95%CI) | *P* |  | OR (95%CI) | *P* |
| rs10830963 | CC/CG/**GG** |  | **1.777 (1.235-2.557)** | **0.002** |  | **1.510 (1.145-1.992)** | **0.003** |  | **1.423 (1.042-1.943)** | **0.026** |
| rs1436953 | CC/CT/**TT** |  | **1.725 (1.134-2.624)** | **0.010** |  | **1.359 (1.057-1.748)** | **0.017** |  | **1.524 (1.024-2.269)** | **0.037** |
| rs7172432 | AA/AG/**GG** |  | **1.609 (1.078-2.403)** | **0.020** |  | **1.394 (1.077-1.805)** | **0.012** |  | 1.363 (0.941-1.975) | 0.100 |
| rs16955379 | **CC**/CT/TT |  | **1.628 (1.009-2.627)** | **0.044** |  | 0.840 (0.654-1.079) | 0.171 |  | 0.642 (0.403-1.023) | 0.061 |
| rs174550 | TT/TC/**CC** |  | 0.775 (0.543-1.105) | 0.159 |  | 0.857 (0.656-1.120) | 0.258 |  | 0.828 (0.607-1.131) | 0.235 |
| rs7041847 | GG/AG/**AA** |  | 1.076 (0.755-1.535) | 0.685 |  | 1.043 (0.785-1.385) | 0.774 |  | 1.057 (0.788-1.418) | 0.711 |
| rs7875253 | **AA**/AC/CC |  | 1.337 (0.832-2.150) | 0.229 |  | 0.831 (0.647-1.066) | 0.144 |  | 0.807 (0.510-1.278) | 0.361 |
| rs7756992 | **GG**/AG/AA |  | 1.002 (0.697-1.440) | 0.993 |  | 0.856 (0.645-1.135) | 0.280 |  | 1.155 (0.852-1.565) | 0.353 |
| rs9465871 | **CC**/CT/TT |  | 0.864 (0.599-1.246) | 0.433 |  | 0.921 (0.693-1.224) | 0.570 |  | 1.301 (0.956-1.769) | 0.093 |
| rs7754840 | GG/CG/**CC** |  | 0.861 (0.594-1.247) | 0.428 |  | 0.883 (0.678-1.150) | 0.357 |  | 0.923 (0.663-1.284) | 0.634 |
| rs3786897 | **AA**/AG/GG |  | 0.842 (0.584-1.216) | 0.359 |  | 0.993 (0.755-1.306) | 0.960 |  | 1.244 (0.906-1.707) | 0.176 |
| rs4376068 | AA/AC/**CC** |  | 1.196 (0.715-2.002) | 0.495 |  | 1.011 (0.785-1.301) | 0.933 |  | 1.205 (0.728-1.997) | 0.467 |
| rs3802177 | **GG**/AG/AA |  | 1.208 (0.838-1.741) | 0.311 |  | 0.813 (0.623-1.062) | 0.129 |  | 0.942 (0.682-1.302) | 0.719 |
| rs13266634 | **CC**/CT/TT |  | 1.170 (0.814-1.683) | 0.396 |  | 0.825 (0.632-1.076) | 0.155 |  | 0.968 (0.702-1.334) | 0.841 |
| rs266729 | **CC**/CG/GG |  | 1.004 (0.602-1.675) | 0.987 |  | 1.129 (0.880-1.449) | 0.340 |  | 0.937 (0.569-1.544) | 0.799 |
| rs290487 | TT/CT/**CC** |  | 1.067 (0.725-1.569) | 0.743 |  | 1.221 (0.945-1.578) | 0.127 |  | 0.934 (0.655-1.333) | 0.707 |

A/a, major allele/minor allele; Risk allele was underlined and in bold.

**Table S5** Clinical characteristics of Chinese women with GDM and controls in the development and validation cohort.

|  | Development cohort | | Validation cohort | |
| --- | --- | --- | --- | --- |
|  | Controls (n=487) | GDM (n=475) | Controls (n=794) | GDM (n=191) |
| Maternal age (years) | 30.69±4.08 | 33.59±4.42^*^ | 30.75±3.90 | 32.01±4.23^†^ |
| Gravidity | 1.90±1.06 | 2.36±1.30^*^ | 1.88±1.08 | 2.04±1.16 |
| 1 | 216 (44.4) | 152 (32.0) ^*^ | 371 (46.7) | 76 (39.8) |
| 2 | 160 (32.9) | 127 (26.7) | 252 (31.7) | 65 (34.0) |
| ≥3 | 111 (22.8) | 196 (41.3) | 171 (21.5) | 50 (26.2) |
| Parity | 0.41±0.54 | 0.57±0.54^*^ | 0.35±0.51 | 0.43±0.55^†^ |
| Nulliparous | 300 (61.6) | 218 (45.9) ^*^ | 533 (67.1) | 113 (59.2) ^†^ |
| Multiparous | 187 (38.4) | 257 (54.1) | 261 (32.9) | 78 (40.8) |
| Height (m) | 1.62±0.05 | 1.60±0.05^*^ | 1.61±0.05 | 1.61±0.05 |
| Pre-pregnancy weight (kg) | 54.51±7.05 | 57.05±9.35^*^ | 54.05±8.00 | 57.15±10.58^†^ |
| Pre-pregnancy BMI (kg/m^2^) | 20.65±2.43 | 22.15±3.29^*^ | 20.81±2.83 | 21.92±3.75^†^ |
| Normal (18.5-24.9 kg/m^2^) | 373 (76.6) | 332 (69.9) ^*^ | 602 (75.8) | 142 (74.3) ^†^ |
| Underweight (<18.5 kg/m^2^) | 87 (17.9) | 53 (11.2) | 142 (17.9) | 23 (12.0) |
| Overweight (≥25 kg/m^2^) | 27 (5.5) | 90 (18.9) | 50 (6.3) | 26 (13.6) |
| Family history of diabetes | 8 (1.6) | 36 (7.6) ^*^ | 27 (3.4) | 19 (9.9) ^†^ |
| Way of conception |  |  |  |  |
| Natural reproduction | 452 (92.8) | 424 (89.3) | 725 (91.3) | 165 (86.4) ^†^ |
| Assisted reproduction | 35 (7.2) | 51 (10.7) | 69 (8.7) | 26 (13.6) |
| OGTT (mmol/L) |  |  |  |  |
| FBG | 4.19±0.31 | 4.74±0.66^*^ | 4.25±0.29 | 4.49±0.50^†^ |
| 1-h BG | 7.39±1.40 | 10.77±1.44^*^ | 7.44±1.24 | 9.89±1.28^†^ |
| 2-h BG | 6.50±1.01 | 9.54±1.41^*^ | 6.50±0.95 | 8.75±1.29^†^ |
| HbA1c (%)^a^ | 4.99±0.27 | 5.25±0.40^*^ | 5.04±0.24 | 5.18±0.30^†^ |
| Insulin levels (μU/ml) |  |  |  |  |
| Fasting insulin^b^ | 7.57±3.01 | 9.56±4.71^*^ | - | - |
| 1-h insulin^c^ | 55.22±31.34 | 69.86±36.58^*^ | - | - |
| 2-h insulin^c^ | 49.69±1.87 | 82.15±44.88^*^ | - | - |

Data were expressed as mean ± SD or number (percentage). OGTT, oral glucose tolerance test; FBG, fasting blood glucose; 1-h BG blood glucose after 1 h; 2-h BG, blood glucose after 2 h; HbA1c, glycosylated hemoglobin. ^*^*P*＜0.05 compared with controls in the development cohort, ^†^*P*＜0.05 compared with controls in the validation cohort.

^a^ HbA1c was compared between 418 controls and 409 GDM in the development cohort, while 780 controls and 190 GDM in the validation cohort.

^b^ Fasting insulin was compared between 191 controls and 153 GDM in the development cohort.

^c^ 1-h insulin and 2-h insulin were compared between186 controls and 150 GDM in the development cohort.

**Table S6** Collinearity diagnosis of four SNPs for GDM in the Chinese population.

| Closest gene | SNP | VIF |
| --- | --- | --- |
| *MTNR1B* | rs10830963 | 1.003 |
| *C2CD4A/B* | rs1436953 | 2.714 |
|  | rs7172432 | 2.712 |
| *CMIP* | rs16955379 | 1.003 |

Collinearity diagnosis of the four loci showed that VIF was all < 3, indicating that there was no collinearity among the four SNPs.

**Figure S1** Evaluation of the model in the test and validation cohort.

(A) ROC for predictive model in test cohort.


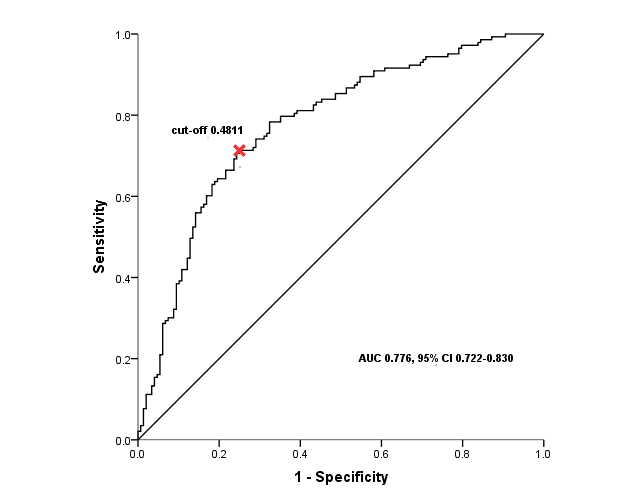


(B) ROC for predictive model in validation cohort.


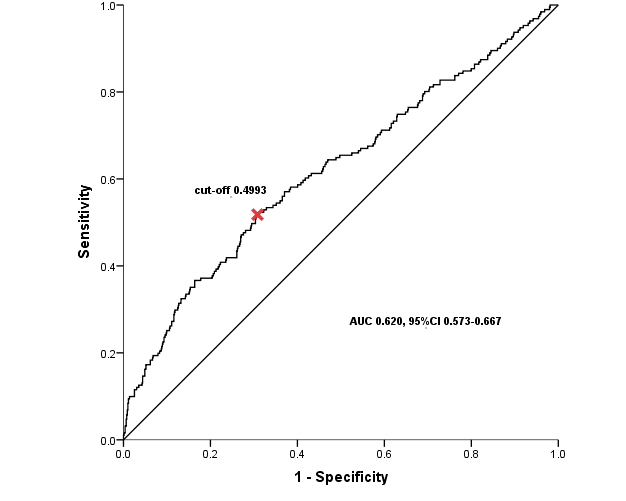


**Reference**

[1] Zhang C, Bao W, Rong Y, et al. (2013) Genetic variants and the risk of gestational diabetes mellitus: a systematic review. Human reproduction update 19(4): 376-390. 10.1093/humupd/dmt013

[2] Kim M, Kim M, Huang L, Jee SH, Lee JH (2018) Genetic risk score of common genetic variants for impaired fasting glucose and newly diagnosed type 2 diabetes influences oxidative stress. Sci Rep 8(1): 7828. 10.1038/s41598-018-26106-z

[3] Mahajan A, Go MJ, Zhang W, et al. (2014) Genome-wide trans-ancestry meta-analysis provides insight into the genetic architecture of type 2 diabetes susceptibility. Nature genetics 46(3): 234-244. 10.1038/ng.2897

[4] Prokopenko I, Langenberg C, Florez JC, et al. (2009) Variants in MTNR1B influence fasting glucose levels. Nature genetics 41(1): 77-81. 10.1038/ng.290

[5] Yamauchi T, Hara K, Maeda S, et al. (2010) A genome-wide association study in the Japanese population identifies susceptibility loci for type 2 diabetes at UBE2E2 and C2CD4A-C2CD4B. Nature genetics 42(10): 864-868. 10.1038/ng.660

[6] Zheng JS, Li K, Huang T, et al. (2017) Genetic Risk Score of Nine Type 2 Diabetes Risk Variants that Interact with Erythrocyte Phospholipid Alpha-Linolenic Acid for Type 2 Diabetes in Chinese Hans: A Case-Control Study. Nutrients 9(4). 10.3390/nu9040376

[7] Imamura M, Shigemizu D, Tsunoda T, et al. (2013) Assessing the clinical utility of a genetic risk score constructed using 49 susceptibility alleles for type 2 diabetes in a Japanese population. The Journal of clinical endocrinology and metabolism 98(10): E1667-1673. 10.1210/jc.2013-1642

[8] Cho YS, Chen CH, Hu C, et al. (2011) Meta-analysis of genome-wide association studies identifies eight new loci for type 2 diabetes in east Asians. Nature genetics 44(1): 67-72. 10.1038/ng.1019

[9] Tarnowski M, Malinowski D, Pawlak K, Dziedziejko V, Safranow K, Pawlik A (2017) GCK, GCKR, FADS1, DGKB/TMEM195 and CDKAL1 Gene Polymorphisms in Women with Gestational Diabetes. Canadian journal of diabetes 41(4): 372-379. 10.1016/j.jcjd.2016.11.009

[10] Ding M, Chavarro J, Olsen S, et al. (2018) Genetic variants of gestational diabetes mellitus: a study of 112 SNPs among 8722 women in two independent populations. Diabetologia 61(8): 1758-1768. 10.1007/s00125-018-4637-8

[11] Muller YL, Piaggi P, Chen P, et al. (2017) Assessing variation across 8 established East Asian loci for type 2 diabetes mellitus in American Indians: Suggestive evidence for new sex-specific diabetes signals in GLIS3 and ZFAND3. Diabetes/metabolism research and reviews 33(4). 10.1002/dmrr.2869

[12] Kwak SH, Kim SH, Cho YM, et al. (2012) A genome-wide association study of gestational diabetes mellitus in Korean women. Diabetes 61(2): 531-541. 10.2337/db11-1034

[13] Peng F, Hu D, Gu C, et al. (2013) The relationship between five widely-evaluated variants in CDKN2A/B and CDKAL1 genes and the risk of type 2 diabetes: a meta-analysis. Gene 531(2): 435-443. 10.1016/j.gene.2013.08.075

[14] Omori S, Tanaka Y, Takahashi A, et al. (2008) Association of CDKAL1, IGF2BP2, CDKN2A/B, HHEX, SLC30A8, and KCNJ11 with susceptibility to type 2 diabetes in a Japanese population. Diabetes 57(3): 791-795. 10.2337/db07-0979

[15] Wu Y, Li H, Loos RJ, et al. (2008) Common variants in CDKAL1, CDKN2A/B, IGF2BP2, SLC30A8, and HHEX/IDE genes are associated with type 2 diabetes and impaired fasting glucose in a Chinese Han population. Diabetes 57(10): 2834-2842. 10.2337/db08-0047

[16] Tabara Y, Osawa H, Kawamoto R, et al. (2009) Replication study of candidate genes associated with type 2 diabetes based on genome-wide screening. Diabetes 58(2): 493-498. 10.2337/db07-1785

[17] Qi Q, Li H, Wu Y, et al. (2010) Combined effects of 17 common genetic variants on type 2 diabetes risk in a Han Chinese population. Diabetologia 53(10): 2163-2166. 10.1007/s00125-010-1826-5

[18] Unoki H, Takahashi A, Kawaguchi T, et al. (2008) SNPs in KCNQ1 are associated with susceptibility to type 2 diabetes in East Asian and European populations. Nature genetics 40(9): 1098-1102. 10.1038/ng.208

[19] Cho YM, Kim TH, Lim S, et al. (2009) Type 2 diabetes-associated genetic variants discovered in the recent genome-wide association studies are related to gestational diabetes mellitus in the Korean population. Diabetologia 52(2): 253-261. 10.1007/s00125-008-1196-4

[20] Chauhan G, Spurgeon CJ, Tabassum R, et al. (2010) Impact of common variants of PPARG, KCNJ11, TCF7L2, SLC30A8, HHEX, CDKN2A, IGF2BP2, and CDKAL1 on the risk of type 2 diabetes in 5,164 Indians. Diabetes 59(8): 2068-2074. 10.2337/db09-1386

[21] Dong F, Zhang BH, Zheng SL, et al. (2018) Association Between SLC30A8 rs13266634 Polymorphism and Risk of T2DM and IGR in Chinese Population: A Systematic Review and Meta-Analysis. Frontiers in endocrinology 9: 564. 10.3389/fendo.2018.00564

[22] Chen G, Xu Y, Lin Y, et al. (2013) Association study of genetic variants of 17 diabetes-related genes/loci and cardiovascular risk and diabetic nephropathy in the Chinese She population. Journal of diabetes 5(2): 136-145. 10.1111/1753-0407.12025

[23] Kasuga Y, Hata K, Tajima A, et al. (2017) Association of common polymorphisms with gestational diabetes mellitus in Japanese women: A case-control study. Endocrine journal 64(4): 463-475. 10.1507/endocrj.EJ16-0431

[24] Bai Y, Tang L, Li L, Li L (2020) The roles of ADIPOQ rs266729 and MTNR1B rs10830963 polymorphisms in patients with gestational diabetes mellitus: A meta-analysis. Gene 730: 144302. 10.1016/j.gene.2019.144302

[25] Chang S, Wang Z, Wu L, et al. (2017) Association between TCF7L2 polymorphisms and gestational diabetes mellitus: A meta-analysis. Journal of diabetes investigation 8(4): 560-570. 10.1111/jdi.12612
